# Orthostatic tremor and its subtypes: a single centre cohort of 74 patients

**DOI:** 10.1007/s00415-026-13625-3

**Published:** 2026-01-27

**Authors:** Aaron Jesuthasan, Solomiia Bandrivska, Lesly Alejandra Colmenares, Leah Jones, Peter G. Bain, Yen F. Tai

**Affiliations:** 1 Department of Neurosciences, Imperial College Healthcare NHS Trust, London, UK; 2 Department of Neurology, Addenbrooke’s Hospital, Cambridge University Hospitals NHS Foundation Trust, Cambridge, UK; 3 Department of Neuromuscular Disorders, UCL Queen Square Institute of Neurology, University College London, London, UK; 4 NHS Lothian, Edinburgh, UK

**Keywords:** Orthostatic tremor, Movement disorders, OT-plus, Pseudo-OT

## Abstract

**Background:**

Orthostatic tremor (OT) is a rare, heterogenous disorder, recently sub-classified into primary OT (isolated 13-18 Hz tremor), OT-plus (OT with additional neurological features) and pseudo-OT (OT with frequencies < 13 Hz). However, to our knowledge no study to date has compared clinical characteristics between all three subgroups.

**Objectives:**

We aim to further define and compare the clinical characteristics of the three different OT subgroups, utilising one of the largest described single centre cohorts to date.

**Methods:**

A retrospective analysis was undertaken of clinical records from 74 OT patients at Charing Cross Hospital between 1999 and 2023, enabling categorisation into subgroups. Clinical characteristics, including treatment efficacy and overall disability, were subsequently described and compared between subgroups.

**Results:**

61 primary OT, 5 OT-plus and 8 pseudo-OT patients were identified. Baseline demographics were comparable between subgroups. Logistic regression suggested age of onset (OR = 1.02, *p* = 0.229), symptom duration (OR = 1.05, *p* = 0.083), tremor frequency (OR = 1.02, *p* = 0.826) and subgroup (OT-plus (OR = 1.86, *p* = 0.565) and pseudo-OT (OR = 1.41, *p* = 0.683)) were not significant predictors of disability. Treatment response varied between subgroup, with primary OT and pseudo-OT patients more frequently reporting symptomatic improvement with clonazepam, gabapentin and/or alprazolam than OT-plus patients.

**Conclusions:**

We provide further insight into the clinical phenotypes of the OT subgroups and encourage future studies to validate these findings with larger sample sizes and establish reliable tools to measure OT severity to better assess disease progression and treatment response.

**Supplementary Information:**

The online version contains supplementary material available at 10.1007/s00415-026-13625-3.

## Introduction

Orthostatic tremor (OT) is defined as a rare tremor disorder that occurs when standing, typically affecting weight-bearing muscles and causes a feeling of unsteadiness or discomfort that disappears when not standing [1]. It tends to affect middle-aged through to elderly individuals, with a female preponderance.

Traditionally, OT was thought to represent a distinct, discrete condition, characterised by high-frequency (13–18 Hz) burst firing in the lower limb and paraspinal muscles, with possible spread to the upper limbs [2]. However, there is now increasing evidence to suggest it is a heterogeneous clinical syndrome encompassing a broader spectrum of features, including cerebellar signs, cognitive impairment and personality disturbance [3].

A sub-classification of OT has consequently been described, with primary OT describing idiopathic cases with an isolated tremor syndrome of 13–18 Hz burst frequency [4–6]. OT-plus syndromes describe patients with OT, who additionally possess other neurological signs, such as parkinsonism, restless legs syndrome, and tardive and orofacial dyskinesias [4, 7]. OT-plus syndromes represent a third of all OT cases and are often attributed to the presence of an alternative neurological disorder, such as aqueduct stenosis, pontine/midbrain lesions, cerebellar degeneration, multiple sclerosis, spontaneous intracranial hypotension, spinal cord lesions, paraneoplastic disorders and peripheral neuropathies [7–12]. A third OT subgroup, pseudo-OT (also termed ‘slow’ OT), has additionally been proposed, denoting OT patients who demonstrate electromyography (EMG) bursts at frequencies ≤ 12 Hz [6, 10]. Pseudo-OT may again be associated with other neurological conditions, such as cerebellar atrophy, multiple sclerosis and Parkinson’s Disease.

Despite the subclassification, the large majority of studies to date have focused solely on primary OT, with very little mentioned about OT-plus syndromes or pseudo-OT [10, 13, 14]. Our report subsequently aimed to address this, utilising one of the largest described single centre OT cohorts to further define and compare the clinical characteristics of the three different OT subgroups.

## Methodology

Clinical records from patients at Charing Cross Hospital who received a diagnosis of OT between 1999 and 2023 were retrospectively examined. 106 patients with a clinical diagnosis of OT were identified; however, 32 patients did not possess available tremor frequency data due to incomplete historical records, external EMG studies without documented frequency, or assessments performed prior to standardized digital EMG archiving, and were therefore excluded from subgroup classification. The remaining 74 patients were divided into primary OT, OT-plus (which also included secondary OT cases with a 13–18 Hz burst frequency) and pseudo-OT subgroups based on the above subclassification. All 106 patients had been evaluated by Movement Disorder specialists (PGB and YFT).

Baseline demographics, initial symptoms at disease onset, delays from symptom onset to formal diagnosis, tremor frequency, comorbidities (including depression, anxiety and other psychiatric conditions) and overall disability were described for the individual subgroups.

Stance disturbance was recorded based on patient-reported symptoms of unsteadiness or discomfort when standing, with relief on sitting or walking. The treatment response of the cohort (as well as the different subgroups) to either medications or deep brain stimulation was explored. Treatment benefit was defined as patient-reported symptomatic improvement sustained for at least three months, as documented in clinical correspondence and follow-up letters. Improvement was typically described in terms of better tolerance or reduced tremor severity when standing. Objective measures such as formal standing time, falls frequency, or standardised activities of daily living scales were not systematically recorded and therefore not included in the assessment of treatment response. Functional disability was defined as the need for one or more of the following: house adjustments, carer support, or walking aids. Logistic regression, using the primary OT subgroup as a reference group, enabled the identification of predictors of disability in OT.

Tremor frequency was investigated by EMG. Patients were assessed in a comfortable upright posture, either seated or standing. Surface EMG electrodes were placed bilaterally on the tibialis anterior and vastus lateralis; in selected cases, wrist flexors and extensors were also recorded. Accelerometers were attached to the lower limbs. Recordings were performed at rest and during co-activation tasks (e.g., tapping or cognitive loading). Neuroimaging reports were reviewed retrospectively, and abnormalities were considered clinically relevant only if they were judged by the treating neurologist to plausibly account for the patient’s orthostatic tremor.

Between-group comparisons were performed using the Kruskal–Wallis test for continuous variables and Fisher’s exact test for categorical variables. Given the small sample sizes of the OT-plus and pseudo-OT subgroups, all subgroup analyses were considered exploratory.

Ethical approval was not required for this study, as this was a retrospective review of case records.

## Results

### Baseline demographic and clinical characteristics

Baseline demographic and clinical characteristics of OT subgroups are summarised in Table 1. Age at symptom onset, age at last assessment, and diagnostic delay were similar across subgroups in exploratory analyses. As expected, tremor frequency differed significantly between subgroups, attributable to the lower frequencies of the pseudo-OT subgroup. Further analyses showed that the 32 patients who did not possess available tremor frequencies and were subsequently excluded also demonstrated a comparable sex distribution, age of symptom onset and diagnosis to the 74 included patients (Supplementary Table 1).

**Table 1:** Baseline demographic and clinical characteristics by orthostatic tremor subgroup

Characteristic	Primary OT (n = 61)	OT-plus (n = 5)	Pseudo-OT (n = 8)	*p*-value
Female sex, n (%)	37 (60.7)	3 (60.0)	5 (62.5)	1.00
Age at last assessment (years), mean ± SD	58.8 ± 12.6	61.4 ± 11.9	60.9 ± 14.8	0.79
Age at symptom onset (years), mean ± SD	49.2 ± 14.8	50.6 ± 13.9	53.4 ± 17.1	0.67
Diagnostic delay (years), median (IQR)	6.0 (2.0–11.0)	6.0 (4.0–9.0)	6.5 (2.0–12.5)	0.94
Tremor frequency (Hz)	15.0 ± 2.7	13.3 ± 2.7	8.1 ± 2.3	< 0.001*

*OT* orthostatic tremor, *IQR* interquartile range

*Significant at *p* ≤ 0.05

### Subgroups

74 OT patients were subdivided into 61 primary OT, 5 OT-plus and 8 pseudo-OT patients (see Supplementary Table 2). The OT-plus subgroup comprised two cases to which the tremor was attributed to neuroleptic use, two cases associated with essential tremor and a further case associated with autism and epilepsy. In the pseudo-OT subgroup, one case was associated with hyperthyroidism, another with neuroleptic use, and one patient had a large-fibre sensory neuropathy. A positive family history for OT was reported by 13 (17.6%) patients in the cohort, including 11 patients in the primary OT (14.9%) and 2 in the pseudo-OT (2.7%) subgroups. All patients who underwent structural neuroimaging (n = 61) had scans, which did not demonstrate a structural abnormality considered causally related to orthostatic tremor (MRI Brain = 57, CT Brain = 4, DaTSCAN = 15, MRI whole spine = 26, MRI cervical spine = 6, MRI lumbar spine = 1). When stratified by subgroup, brain and/or spinal imaging had been performed in the majority of primary OT, OT-plus, and pseudo-OT patients. Mild or non-specific findings, such as age-related white matter hyperintensities or degenerative spinal changes without cord compression or myelopathy, were considered incidental and classified as normal for the purposes of this study.

### Age and sex

**Fig. 1:**
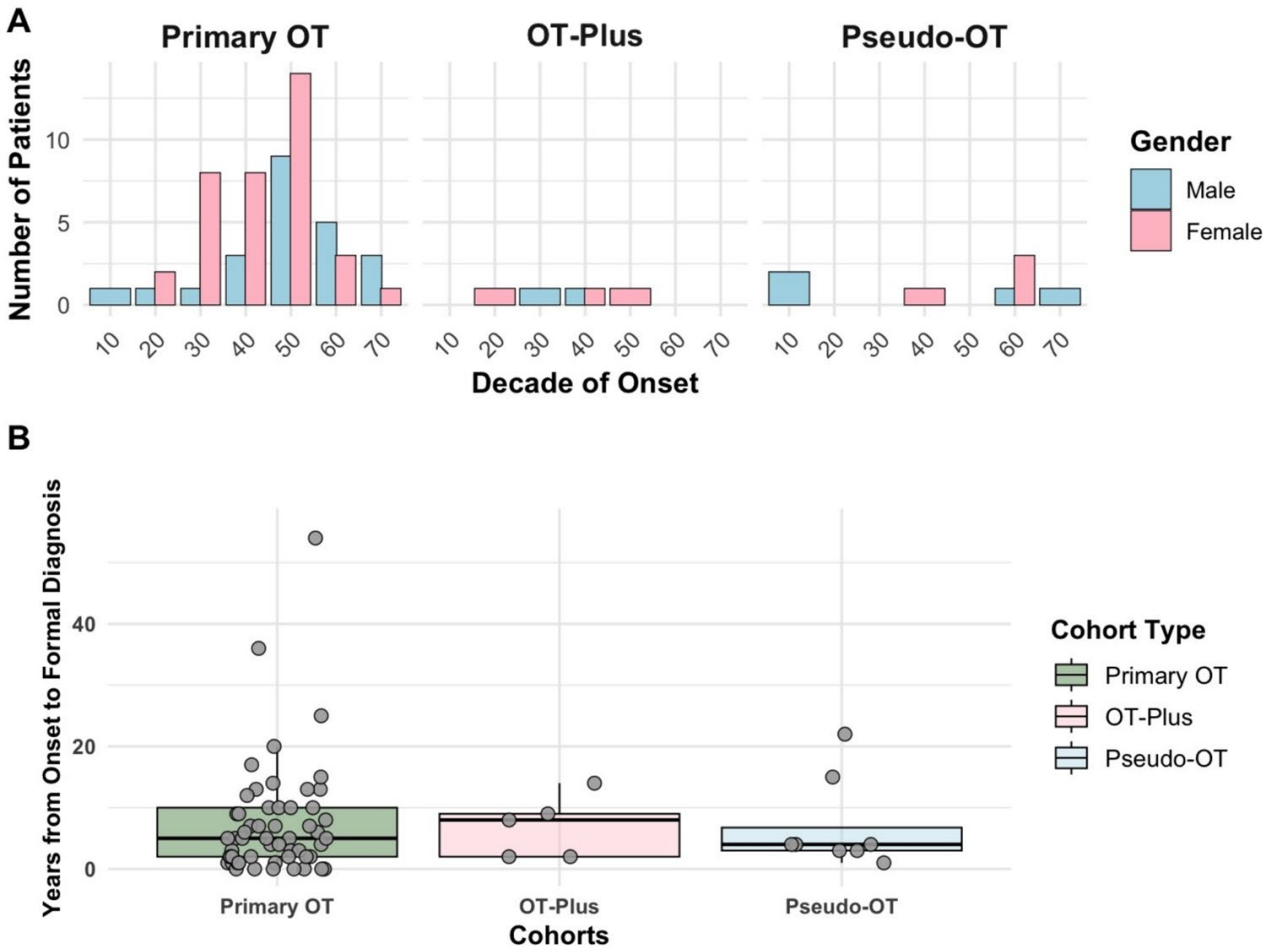
**A** Age of disease onset in the cohort and (**B**) years from disease onset to formal diagnosis of orthostatic tremor (box represents interquartile range and central line denotes the median value). OT, Orthostatic tremor

The cohort demonstrated a female predominance, representing 60.8% (45/74) of cases. The mean age at symptom onset and diagnosis was 49.7 years (SD = 15.0, IQR = 19.0) and 56.5 years (SD = 13.4, IQR = 16.0) respectively. The age of disease onset for the cohort is shown in Fig. 1a and varied by subgroup: primary OT and OT-plus peaked in the 6th and 5th decades respectively, whereas pseudo-OT appeared to demonstrate a bimodal pattern with patients presenting in their 2nd as well as 7th decades of life. On average, a delay from initial symptom onset to formal diagnosis was 7.4 years in the cohort (range = 0–54 years): 7.3 years (SD = 9.3) for primary OT, 6.8 years (SD = 4.6) for OT-plus, and 7 years (SD = 7.4) for pseudo-OT (see Fig. 1b).

### Symptoms and examination findings

The initial symptoms at disease onset for the cohort are summarised in Table 2. Stance disturbance was the most common initial symptom, followed by leg tremor.

**Table 2:** Initial symptom at disease onset divided by OT subgroup

Symptoms at onset	Primary OT (n = 61)	OT-plus (n = 5)	Pseudo-OT (n = 8)
Stance disturbance	32	1	-
Tremor in legs	18	2	3
Stance disturbance with tremor in legs	2	2	2
Gait disturbance	3	–	1
Balance problems	2	–	1
Pain (unspecified)	2	–	–
Stance disturbance with tremor in legs and imbalance	1	–	–
Tremor in hands and legs	–	1	–
Asymmetrical tremor in one leg	1	–	–
Bilateral leg weakness	–	–	1
Abdominal tremor	1	–	–

*OT* Orthostatic tremor

Ultimately, as the disease progressed a symmetrical lower limb postural tremor was present in all, but one case (who presented with an asymmetrical left leg tremor) (Table 3). Difficulty standing became an issue for 63 patients (50/61 primary OT, 5/5 OT-plus, 8/8 pseudo-OT) as the disease progressed. Gait impairment affected 15 patients. Forty additionally exhibited a symmetrical upper limb postural tremor, five presented with head tremor, four with a voice tremor and two with a face tremor.

**Table 3:** Number of patients in each subgroup with additional neurological signs as the disease progressed

Neurological signs and symptoms during the course of the disease	Primary OT (n = 61)	OT-plus (n = 5)	Pseudo-OT (n = 8)
Lower limb postural tremor: Symmetric Asymmetric	60 1	5 0	8 0
Upper limb postural tremor	31	4	5
Abnormal Handwriting	11	0	1
Difficulty Standing	50	5	8
Gait impairment	13	0	2
Head Tremor	3	1	1
Voice Tremor	3	1	0
Face Tremor	2	0	0
Abnormal Eye Movements	1	0	0
Imbalance	3	0	1
Incoordination	0	0	1
History of falls	0	0	1

*OT* Orthostatic tremor

### Disability and comorbidities

33/74 patients (44.6%) met the predefined disability criterion, defined as requiring at least one of the following: walking aids, carer support, or home adaptations. Disabled patients were older at the time of last clinical assessment compared with non-disabled patients (mean age 64.8 vs 55.1 years). In the cohort, 14 patients had left work, 33 required walking aids and 24 required assistance with personal care due to OT-related symptoms (see Supplementary Table 2). In exploratory logistic regression analysis (33 events), age at onset, symptom duration, tremor frequency, and OT subgroup did not demonstrate a statistically robust association with disability; however, confidence intervals were wide, reflecting limited power (Supplementary Table 3).

**Fig. 2:**
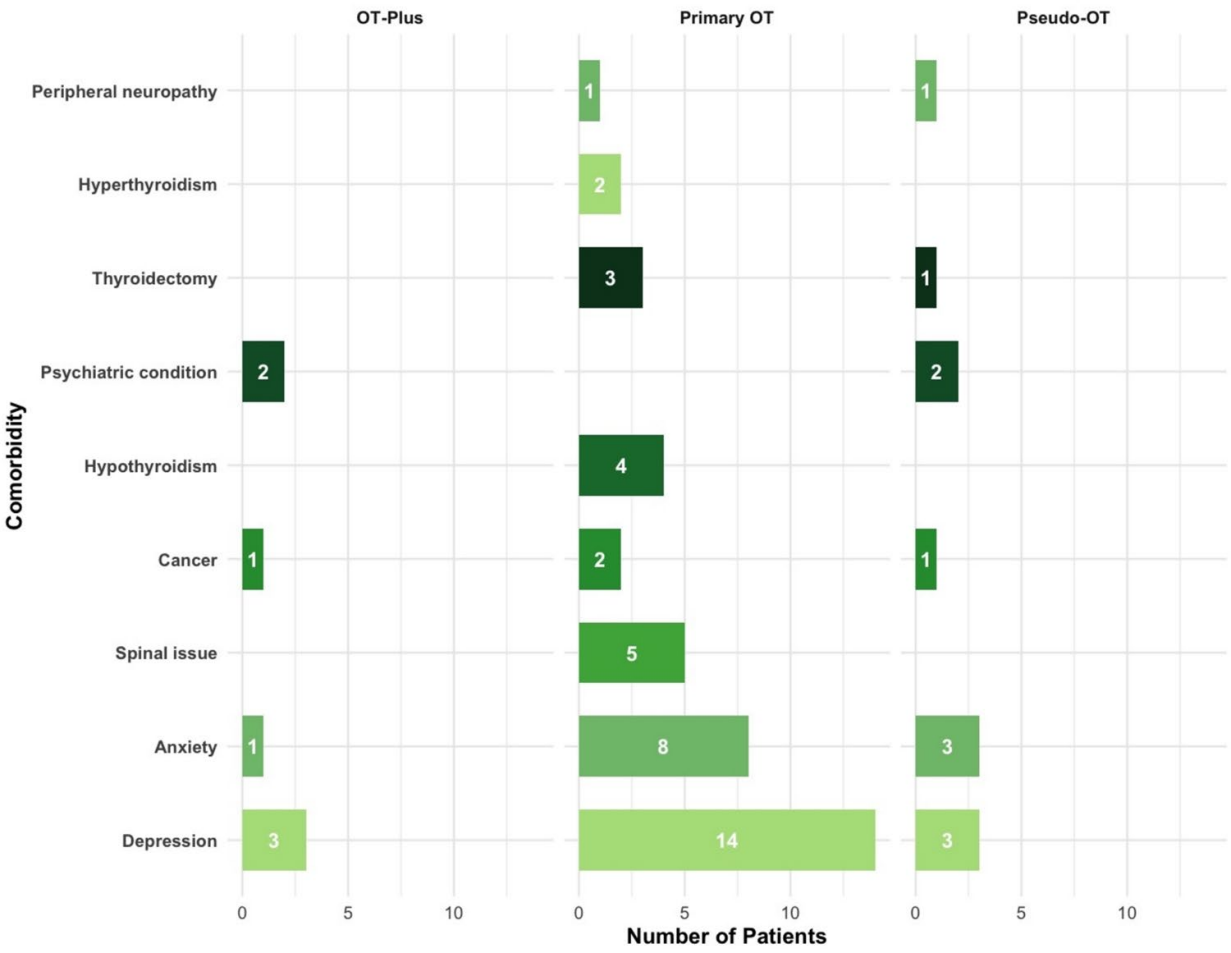
Comorbidities of individual subgroups. OT, Orthostatic tremor

Depression and anxiety followed by peripheral neuropathy, spinal pathology (without myelopathy or cord compression) and thyroid disorders were the most commonly reported comorbidities across all three subgroups (Fig. 2). Rates of depression and/or anxiety did not differ significantly between subgroups in exploratory analyses (primary OT 39.3%, OT-plus 40.0%, pseudo-OT 50.0%; Fisher’s exact test, *p* = 0.86). However, psychiatric comorbidity was numerically more common in disabled compared with non-disabled patients (54.5% vs 29.3%), although this did not reach statistical significance (*p* = 0.06). Alcohol consumption and smoking were reported in 15 and 20 individuals respectively.

### Treatment efficacy

Table 4 summarises trialled treatments, including medications and deep brain stimulation, as well as their efficacy in relieving tremor across subgroups.

**Table 4:** Medications attempted and their treatment efficacy across subgroups

Medication	Primary OT (n = 61) Effective/Attempted (%)	OT-plus (n = 5) Effective/Attempted (%)	Pseudo-OT (n = 8) Effective/Attempted (%)
Alprazolam	3/11 (27.3)	0/2	2/3 (66.7)
Amantadine	0/1	–	–
Amitriptyline	2/7 (28.6)	0/1	0/1
Benzhexol	0/1	–	–
Buspirone	0/1	–	–
Clonazepam	18/41 (43.9)	0/5	2/4 (50.0)
Diazepam	1/2 (50.0)	–	–
Fluoxetine	0/1	–	–
Gabapentin	11/29 (37.9)	1/2 (50.0)	0/1
Levetiracetam	0/1	–	–
Levodopa	0/12	–	0/1
Mirtazapine	0/2	0/2	–
Perampanel	0/1	0/1	–
Phenobarbitone	0/2	–	–
Pramipexole	0/1	–	0/1
Pregabalin	1/6 (16.7)	0/2	–
Primidone	3/19 (15.8)	0/3	0/1
Propranolol	1/3 (33.0)	0/3	1/3 (33.3)
Quetiapine	0/1	–	–
Ropinirole	0/2	–	0/1
Rotigotine	0/1	–	–
Topiramate	1/4 (25.0)	–	0/1
Trihexyphenidyl	0/1	–	–
VIM/ZI DBS	–	1/1 (100.0)	–

*DBS* deep brain stimulation, *OT* Orthostatic tremor, *VIM* ventral intermediate nucleus, *ZI* zona incerta

Clonazepam, gabapentin, alprazolam and primidone were the most frequently attempted medications in the cohort. Clonazepam was typically prescribed at doses ranging from 0.25 −2 mg/day, gabapentin 300–1800 mg/day, alprazolam 0.25–1.5 mg/day, and primidone 62.5–250 mg/day, titrated according to tolerability and perceived benefit. Treatments were generally trialled sequentially as monotherapy, reflecting routine clinical practice, with limited use of combination therapy. Medication trials were frequently discontinued due to lack of efficacy or adverse effects, including sedation, dizziness, and gait instability. One OT-plus patient who underwent bilateral deep brain stimulation targeting the ventral intermediate nucleus and zona incerta for co-existing essential tremor subsequently reported marked improvement in orthostatic tremor symptoms.

## Discussion

We describe, to our knowledge, the first study that describes the clinical characteristics of the three OT subgroups: primary OT, OT-plus and pseudo-OT, utilising a single centre cohort.

Our cohort demonstrated similar demographical characteristics to previous investigations, supporting a female and middle-aged predominance to OT. We re-emphasise that there is often a significant delay in the diagnosis of OT, amounting to approximately 7 years in all of our subgroups [7, 15]. Rigby et al. (2015) reported a shorter delay in diagnosis in pseudo-OT compared to primary OT, potentially due to more disabling symptoms and earlier specialist referral, however we failed to replicate this in our study [16]. The diagnostic delay in OT is likely to be multifactorial, predominantly due to the gradual onset and slow progression of symptoms that primarily affect specific daily living activities, such as standing [17]. Secondly, given its rarity in practice, OT is often neglected or misdiagnosed by clinicians until the symptoms become sufficiently apparent to prompt a specialist referral [18].

Stance disturbance and tremor were noted to be the most common initial symptoms in all of our subgroups, with difficulty standing being ultimately noted in almost the entirety of the cohort by the time of presentation to a clinician. This compares to a smaller study by Bicart-Sée et al. (2021), involving 10 primary OT and 17 pseudo-OT patients, in which only 30% of primary OT patients presented with a movement disorder at first visit whilst 100% of pseudo-OT patients displayed extrapyramidal or cerebellar disorders [19]. However, over the disease trajectory we show that OT is indeed a largely progressive and clinically heterogenous condition, with the eventual development of other symptoms external to the leg tremor and also commonly spread of the tremor to the upper limbs [3, 20]. This contests previous suggestions from Gerschlager et al. (2004) amongst other smaller studies, who reported a stable disease course in the majority of OT cases and only a small proportion of patients develop proximal spread of their tremor [7, 21, 22]. A study by Ganos et al. (2016) supports our findings, demonstrating with long-term follow-up of their OT patients that 80–90% suffered worsening of their symptoms and 24% experienced falls [23].

We also studied functional disability in our cohort, noting that, unlike essential tremor in which tremor frequency is suggested to have a significant impact on overall disability, this did not seem to be the case for OT. We additionally did not find a link between age of disease onset and functional disability. Symptom duration, whilst showing a trend toward higher odds of overall disability in keeping with a previous case series of 68 OT patients, did not reach statistical significance [23]. The potential contribution of body mass index to disability in OT remains unclear and represents an area for future prospective investigation, particularly in light of recent data linking BMI to disease severity and functional burden in essential tremor [24].

Psychiatric comorbidity may also contribute to perceived disability in orthostatic tremor. Anxiety and depression were common across all OT subgroups in our cohort and have previously been shown to substantially impair health-related quality of life in OT patients [25]. Maugest et al. (2018) further demonstrated that fear of falling is among the strongest predictors of reduced quality of life in this population. Spinal pathology and thyroid dysfunction were also frequently reported in our cohort; however, these were considered incidental and unlikely to be causally related to orthostatic tremor [18, 24].

Our findings reaffirm the difficulty in treating OT, as even partial symptomatic management of the disease often occurs after trials of several medications. However, we recognise that interpretation of treatment efficacy is limited by the retrospective design and reliance on patient-reported outcomes in our study, and should therefore be considered descriptive rather than definitive. Of the available treatment options, clonazepam, gabapentin and alprazolam remain the most commonly trialled and successful medications, while observations with other seldom used medications remain mainly anecdotal [7, 20, 21, 26]. There may be a slight disparity between the efficacy of treatments between subgroups, with the OT-plus subgroup perhaps being more difficult to treat, however this is based on small subgroup sample sizes and requires validation with larger cohorts.

Surgical treatment with DBS was attempted in only one (OT-plus) patient from our cohort for essential tremor rather than OT, targeting the bilateral VIM and ZI. This resulted in a significant improvement in their OT symptoms, similar to studies with larger numbers of OT patients who underwent DBS [15, 20, 27, 28]. However, DBS remains largely reserved for medication-resistant or intolerant OT patients; hence most patients undergo surgery only after years of symptoms. It subsequently remains unknown if earlier intervention with DBS may translate to greater success in relieving symptoms.

We acknowledge several limitations to our study. Firstly, as this was a retrospective study, we could not ensure that the same operators and equipment were used to perform the objective tremor assessments in all patients. Second, given the absence of established tools to assess OT severity and associated disability, an accurate examination of disease progression and treatment response was based solely on subjective patient reporting. This is undoubtedly susceptible to placebo effects and recall bias, and is similar to other previously reported OT case series [17, 24]. Future studies should subsequently aim to construct specific rating scales to capture changes in the different characteristics of OT, such as tremor-onset latency, standing time, standing ADLs, medication use, global impression of change, and gait aid use to facilitate better assessment of disability and treatment response [28]. Approximately one third of the original cohort was excluded due to missing EMG frequency data, which may have introduced selection bias toward more recent or more typical OT cases. However, excluded patients demonstrated similar demographical characteristics to the analysed cohort, partially mitigating this concern. We also acknowledge the sample sizes of our subgroups, particularly OT-plus and pseudo-OT, are small, especially when commenting upon treatment efficacy. This limited the ability to perform more detailed statistical, comparative analyses between subgroups in our study. Larger-scale studies, likely requiring multicentre collaboration, are therefore required to validate our findings. Furthermore, the choice of medical treatment in our investigation was based on the treating neurologists’ preference and experience, reflecting real-life neurology practice rather than randomised, blinded comparisons. Certain medications, such as clonazepam, which are generally considered to be more efficacious in OT were often hence used as a first line option. This likely introduced bias into the treatment response reported here.

Finally, an important issue highlighted by our findings are the considerable similarities across many studied aspects, especially between primary OT and OT-plus patients. This subsequently raises the question as to whether the current distinction between these two subgroups is clinically useful in everyday practice. This is further emphasised by the relatively vague consensus definition for OT-plus, which defines it as primary OT in combination with other neurological conditions [6], without further characterisation into which conditions should particularly be included. For example, if a patient presents with OT in combination with a common, incidental neurological condition (e.g. migraine, tension headache, carpal tunnel syndrome) this would still be classified as OT-plus despite the two likely being unrelated. Similarly, if a patient has OT and then develops a condition later on in life, such as Alzheimer’s disease, this would infer that they have migrated from primary OT to OT-plus. This further blurs the separation between the two subgroups. We therefore propose that OT may simply describe a specific symptom and sign that can occur in isolation or with other neurological and medical conditions. Future studies utilising larger, ideally multicentre cohorts are required to validate this, to further reinforce a need for revision of the current OT consensus definitions.

## Conclusion

Our investigation suggests that primary OT, OT-plus, and pseudo-OT patients present in similar clinical fashion, with stance disturbance and leg tremor tending to be the most common initial symptoms. However, patients often during their disease course develop further symptoms, including spread of their tremor, which can be disabling. There remains a significant need for good quality data, utilising randomised double-blind placebo-controlled trials, to guide physicians on the treatment for OT as well as its subgroups to improve the current trial and error approach with medications. This is particularly the case, as medications presently produce modest and temporary benefits at best. Furthermore, larger scale studies, likely requiring multicentre collaboration, are encouraged to validate promising treatments, including the use of DBS.

## Supplementary Information

Below is the link to the electronic supplementary material.

Supplementary file1 (DOCX 15 KB)

Supplementary file2 (XLSX 36 KB)

Supplementary file3 (DOCX 15 KB)

## Data Availability

The authors declare that data supporting the findings of this study are available within the article.
