# Supplementary material for: Orthostatic tremor and its subtypes: a single centre cohort of 74 patients
Source: J Neurol. 2026 Jan 27;273(2):100. doi: 10.1007/s00415-026-13625-3 (PMC12847164; doi:10.1007/s00415-026-13625-3)
Supplement: Supplementary file 1 — Supplementary file1 (DOCX 15 KB) [file 415_2026_13625_MOESM1_ESM.docx]

## **Supplementary Table 1. Baseline characteristics of included and excluded OT patients**

| **Characteristic** | **Included (EMG frequency available) n = 74** | **Excluded (EMG frequency unavailable) n = 32** |
| --- | --- | --- |
| **Sex, n (%)** |  |  |
| Female | 45 (60.8%) | 20 (62.5%) |
| Male | 29 (39.2%) | 12 (37.5%) |
| **Age at symptom onset (years)** |  |  |
| Mean ± SD | 49.7 ± 15.0 | 53.1 ± 18.1 |
| Median (IQR) | 52.0 (42.0–61.0) | 55.5 (44.0–66.3) |
| **Age at diagnosis (years)** |  |  |
| Mean ± SD | 56.5 ± 13.4 | 61.9 ± 16.2 |
| Median (IQR) | 58.0 (48.0–64.0) | 65.0 (56.5–73.5) |
| **Clinician-assigned OT subtype*** |  |  |
| Primary OT | 61 (82.4%) | Not classifiable |
| OT-plus | 5 (6.8%) | Not classifiable |
| Pseudo-OT | 8 (10.8%) | Not classifiable |

**Abbreviations:** OT, orthostatic tremor; EMG, electromyography; IQR, interquartile range; SD, standard deviation.

*Formal OT subgroup classification was not possible in excluded patients due to missing EMG frequency data.
