# Supplementary material for: Orthostatic tremor and its subtypes: a single centre cohort of 74 patients
Source: J Neurol. 2026 Jan 27;273(2):100. doi: 10.1007/s00415-026-13625-3 (PMC12847164; doi:10.1007/s00415-026-13625-3)
Supplement: Supplementary file 3 — Supplementary file3 (DOCX 15 KB) [file 415_2026_13625_MOESM3_ESM.docx]

### **Supplementary Table 3. Logistic regression (exploratory)**

| **Predictor** | **OR** | **95% CI** | **p value** |
| --- | --- | --- | --- |
| Age at onset (per year) | 1.02 | 0.98–1.06 | 0.23 |
| Symptom duration (per year) | 1.05 | 0.99–1.11 | 0.08 |
| Tremor frequency (per Hz) | 1.02 | 0.86–1.20 | 0.83 |
| OT-plus vs primary OT | 1.86 | 0.22–15.6 | 0.57 |
| Pseudo-OT vs primary OT | 1.41 | 0.29–6.95 | 0.68 |

**Abbreviations:** CI, Confidence interval; OR, Odds ratio; OT, Orthostatic tremor.
